# Influence of Mckenzie protocol and two modes of endurance exercises on health-related quality of life of patients with long-term mechanical low-back pain

**DOI:** 10.11694/pamj.supp.2014.17.1.2950

**Published:** 2014-01-18

**Authors:** Chidozie Emmanuel Mbada, Olusola Ayanniyi, Samuel Olusegun Ogunlade, Elkanah Ayodele Orimolade, Ajibola Babatunde Oladiran, Abiola Oladele Ogundele

**Affiliations:** 1 Department of Medical Rehabilitation, College of Health Sciences, Obafemi Awolowo University, Ile-Ife, Nigeria; 2 Department of Physiotherapy, College of Medicine, University of Ibadan, Ibadan, Nigeria; 3 Obafemi Awolowo University Teaching Hospitals Complex, Ile-Ife, Nigeria

**Keywords:** Mckenzie protocol, endurance exercises, quality of life, back pain

## Abstract

**Introduction:**

Long-term Mechanical Low-Back Pain (LMLBP) negatively impacts on patients’ physical capacity and quality of life. This study investigated the relationship between Health-Related Quality of Life (HRQoL) and pain intensity, and the influence of static and dynamic back extensors’ endurance exercises on HRQoL in Nigerian patients with LMLBP treated with the McKenzie Protocol (MP).

**Methods:**

A single-blind controlled trial involving 84 patients who received treatment thrice weekly for eight weeks was conducted. Participants were assigned to the MP Group (MPG), MP plus Static Back Endurance Exercise Group (MPSBEEG) or MP plus Dynamic Endurance Exercise Group (MPDBEEG) using permuted randomization. HRQoL and pain was assessed using the Short-Form (SF-36) questionnaire and Quadruple Visual Analogue Scale respectively.

**Results:**

Sixty seven participants aged 51.8 ± 7.35 years completed the study. A total drop-out rate of 20.2% was observed in the study. Within-group comparison across weeks 0-4, 4-8 and 0-8 of the study revealed significant differences in HRQoL scores (p < 0.05). Treatment Effect Scores (TES) across the groups were significantly different (p = 0.001). MPSBEEG and MPDBEEG were comparable in TES on General Health Perception (GHP) at week 4; and GHP and Physical Functioning at week 8 respectively (p > 0.05). However, MPDEEG had significantly higher TES in the other domains of the SF-36 (p = 0.001).

**Conclusion:**

HRQoL in patients with LMLBP decreases with pain severity. Each of MP, static and dynamic back extensors endurance exercises significantly improved HRQoL in LMLBP. However, the addition of dynamic back extensors endurance exercise to MP led to greater improvement in HRQoL.

## Background

Low-Back Pain (LBP) is described as the constellation of symptoms of pain or discomfort originating from impairments in the structures in the low back [1–2]. LBP is one of the most common ailments afflicting mankind [3]. It is a complicated condition which affects the physiological and psychosocial aspects of the patient [4, 5]. Epidemiological reports indicate that 70 to 85% of all people have LBP at some time in their life [1, 6]. The World Health Organization predicted that the greatest increases in LBP prevalence in the next decade will be in developing nations [7]. In line with this, a systematic review by Louw et al [8] concluded that the global burden and prevalence of LBP among Africans is rising.

It is estimated that 80-90% of patients with LBP will recover within six weeks, regardless of treatment [9]. However, 5-15% of all people that have LBP will develop long-term LBP (i.e. LBP of 12 weeks and longer) [10, 11]. The patient subgroup with long-term LBP accounts for 75-90% of the socioeconomic cost of LBP [12] and over 30% of these patients with long-term LBP seek healthcare for their back complaints. Long-term LBP significantly impacts on patients’ physical [13], psychological and social functioning [14] and can affect well-being and quality of life [15]. Reduced quality of life in patients with long-term LBP is associated with poor prognosis [16], intermittent or recurrent episodes of LBP [17], disability [18] and psychosocial dysfunction [19, 20].

Assessment of Health-Related Quality of Life (HRQoL) in relation to LBP has been recommended in LBP management [21, 22]. Several HRQoL instruments have been developed to assess self-perceived general health status [21, 22]. The SF-36 Health Status Questionnaire, though a generic instrument, has been recommended in the assessment of HRQoL of patients with long-term LBP [22] and it assesses eight domains such as physical functioning, role limitations due to physical problems, bodily pain, general health perceptions, vitality, social functioning, role limitation due to emotional problems and general mental health [23, 24].

Consequent to the foregoing, treatment intervention that may help improve the HRQoL of patients with long-term LBP has been advocated. Although, physiotherapy plays an important role in the management of patients with LBP, the traditional approach based on biomedical model, which is centered on the treatment of impairments and patho-physiological variables, may not fully addressed the wider range of factors including psychosocial impairments associated with long-term LBP [25, 26]. However, long-term LBP is considered to be a multi-factorial bio-psychosocial problem which has an impact on both social life [27, 28] and quality of life [29] and thus requires a multi-dimensional approach based on a bio-psychosocial model (a model that includes physical, psychological and social elements) in its assessment and treatment [30, 31].

Based on empirical recommendations from research, recent decades have witnessed tremendous advances in preventive, pharmacological and physiotherapy management for a limited number of patients with LBP especially in developed countries. However, the improvement in health outcomes observed in most Western countries over the past few decades has not been achieved in Africa [32] and therefore, the health of Africans is of global concern [8]. Compared with Australians [33], Europeans [34] and North Americans [35], the use of exercise as medicine in Africans is poor. Exercise is the central element in the physical therapy management of patients with long-term LBP [9, 36]. Exercise often does not require expensive instruments and probably the cheapest intervention and one in which the patient has some measure of direct control [37]. Nonetheless, it remains inconclusive which exercise regimen will significantly influence the quality of life of patients with long-term LBP. The McKenzie Protocol (MP) is one of the most commonly used physical therapy interventions in long-term mechanical LBP with documented effectiveness [38–41]. However, there is a dearth of studies that have investigated the influence of the MP on HRQoL in patients with long-term mechanical LBP. Therefore, this study was intended to answer the following questions: (1). Will pain intensity significantly influence HRQoL? (2) Will static and dynamic back extensors’ endurance exercises significantly influence HRQoL in Nigerian patients with long-term mechanical LBP (LMLBP) treated with the MP?

## Methods

Eighty four patients with LMLBP participated in this single-blind randomized trial. The participants were consecutively recruited from the physiotherapy department, Obafemi Awolowo University (OAU) Teaching Hospitals Complex and the OAU Health Centre, Ile-Ife, Nigeria. The McKenzie Institute's Lumbar Spine Assessment Format (MILSAF) [3] was used to determine eligibility to participate in the study. Based on the MILSAF, patients who demonstrated Directional Preference (DP) for extension only were recruited to ensure homogeneity of samples. DP is described as the posture or movement that reduces or centralizes radiating pain that emanates from the spine. Exclusion criteria were red flags indicative of serious spinal pathology with signs and symptoms of nerve root compromise (with at least two of dermatomal sensory loss, myotomal muscle weakness and reduced lower limb reflexes), individuals with any obvious spinal deformity or neurological disease; pregnancy; previous spinal surgery; previous experience of static and dynamic endurance exercise and having DP for flexion, lateral or no DP. Long-term low-back pain was defined as a history of LBP of not less than 3 months [42].

Based on the sample size table by Cohen [43] with alpha level set at 0.05, degree of freedom at 2, effect size at 0.25, and power at 80, the study found a minimum sample size of 52. However, in order to accommodate for possible attrition or loss during the study, a total of 75 patients (25 per group) was included. The participants were randomly assigned to one of three treatment groups using permuted block randomization; the McKenzie Protocol (MP) Group (MPG) (n = 29), MP plus Static Back Endurance Exercise Group (MPSBEEG) (n = 27) and MP plus Dynamic Back Endurance Exercise Group (MPDBEEG) (n = 28). Sixty seven (32 males (47.8%) and 35 females (52.2%) participants completed the eight week study. Twenty five participants completed the study in MPG, 22 in MPSBEEG and 20 in MPDBEEG. A total drop-out rate of 20.2% was observed in the study. Fourteen percent of participants in MPG were lost to follow-up. Nineteen percent of the participants in MPSBEEG dropped out (out of these, 40% were lost to follow-up while 60% absconded due to improvement in their health condition). In the MPDBEEG, 28.6% of the participants dropped out (37.5% were lost to follow-up while 62.5% absconded due to improvement in their health condition).

Treatment was given thrice weekly for eight weeks and outcomes were assessed at the end of the fourth and eighth week of study. Ethics and Research Committee of the Obafemi Awolowo University Teaching Hospitals Complex and the joint University of Ibadan /University College Hospital Institutional Review Committee respectively gave approval for the study.

### Instruments

A height meter calibrated from 0-200cm was used to measure the height of each participant to the nearest 0.1cm. A weighing scale was used to measure the body weight of participants in kilograms to the nearest 1.0Kg. It is calibrated from 0 - 120kg. A metronome (Wittner Metronom system Maelzel, Made in Germany) was used to set a uniform tempo for dynamic back endurance muscles endurance test, which involves repeated contraction or movements over a period of time performed synchronously to the metronome beat. Patients lay on a plinth for the MP, static and dynamic back endurance exercise respectively.

General Health Status Questionnaire - Short Form -36 (SF-36) was used to assess the quality of life of the participants. The SF-36 has been recommended in the assessment of patients with long-term LBP [24, 44, 45]. A Yoruba translated version of the Health Status Questionnaire (SF-36) was used for participants who were literate in the Yoruba language and preferred the Yoruba version. The translation was done at the department of linguistics and African languages of Obafemi Awolowo University, Ile Ife. Pearson product moment correlation coefficient (r) of 0.84 was obtained for the criterion validity of the back translation of the Yoruba version. Quadruple Visual Analogue Scale (QVAS) was used to assess pain intensity of participants. QVAS is a reliable and valid method for pain measurement [46, 47]. A Yoruba translated version of the QVAS was used for participants who were literate in the Yoruba language and prefers the Yoruba version. The translation was done at the department of linguistics and African languages of Obafemi Awolowo University, Ile Ife. Pearson product moment correlation coefficient (r) of 0.88 was obtained for the criterion validity of the back translation of the Yoruba version.

### Treatment

Treatment for the different groups (MPG, MPSBEEG and MPDBEEG) comprised three phases including warm up, main exercise and cool down. Prior to treatment, the participants were instructed in details on the study procedures. This was followed by a low intensity warm-up phase of five minutes duration comprising active stretching of the upper extremities and low back and strolling at self-determined pace around the research venue. Treatment also ended with a cool-down phase comprising of the same low intensity exercise as the warm-up for about five minutes.

The McKenzie Protocol (MP) involved a course of specific lumbosacral repeated movements in extension that cause the symptoms to centralize, decrease or abolish. The determination of the direction preference for extension was followed by the main MP activities including “Extension lying prone”, “Extension In Prone” and “Extension in standing”. The MP also included a set of back care education instructions which comprised a 9 item instructional guide on standing, sitting, lifting and other activities of daily living for home exercise for all the participants (Appendix).

In addition to completing the MP (i.e., back extension exercises plus the back care education), static back extensors endurance exercise which included five different static exercises differentiated by the alteration of the positions of the upper and lower limbs with the patient in prone lying on a plinth was carried out [48]. The participants began the exercise training programme with the first exercise position, but progressed to the next exercises at their own pace when they could hold a given position for 10 seconds. On reaching the fifth progression, they continued with the fifth progression until the end of the exercise programme [48, 49]. The following were the five exercise progressions:

1. Participant lay in prone position with both arms by the sides of the body and lifting the head and trunk off the plinth from neutral to extension;
2. Participant lay in prone position with the hands interlocked at the occiput so that shoulders were abducted to 90° and the elbows flexed, and lifting the head and trunk off the plinth from neutral to extension;
3. Participant lay in prone position with both arms elevated forwards, and lifting the head, trunk and elevated arms off the plinth from neutral to extension;
4. Participant lay in prone position and lifting the head, trunk and contralateral arm and leg off the plinth from neutral to extension; and
5. Participant lay in prone position with both shoulders abducted and elbows flexed to 90°, and lifting the head, trunk and both legs (with knees extended) off the plinth.

If pain was aggravated during the exercise, the participant was asked to stop. If the pain diminished within 5 minutes after the exercise, he/she was asked to continue the exercise but to hold the exercise position for only 5 seconds. The participant was asked to progress to 10 seconds if there was no adverse response. Each exercise was repeated 9 times. After 10 repetitions, the participant was instructed to rest for between 30 seconds to 1 minute. Static holding time in the exercise position was gradually increased to 20 seconds to provide a greater training stimulus [50, 51]. The dosage of series of 10 repetitions was adopted from a previous protocol for participants with sub-acute LBP [52].

In addition to completing the MP, dynamic back extensors endurance exercise which included five different isokinetic exercises differentiated by the alteration of the positions of the upper and lower limbs with the patient in prone lying on a plinth was carried out. The dynamic back endurance exercise was an exact replica of the static back extensors endurance exercise protocol in terms of exercise positions, progressions and duration. However, instead of static posturing of the trunk in the prone lying position and holding the positions of the upper and lower limbs suspended in the air during all the five exercise progressions for the 10 seconds, the participant was asked to move the trunk and the suspended limbs 10 times.

If pain was aggravated during the exercise, participant was asked to stop. If the pain diminished within 5 minutes after the exercise, the participant was asked to continue the exercise but to carry out only 5 movements in the exercise position. The participant was asked to progress to 10 movements if there is no adverse response. Each exercise was repeated 9 times. After 10 repetitions, the participants were instructed to rest for between 30 seconds to 1 minute. The number of movements of the trunk in the exercise position was gradually increased to 20 seconds to provide a greater training stimulus.

In order to achieve adequate training effect based on recommendation of previous studies, a 30 to 45 minute exercise duration, thrice weekly and eight weeks exercise; and training load of 10 seconds static hold or 10 repetitions per exercise position was adopted [53, 54].

The researchers (CEM and OA) were credentialed in the McKenzie method and supervised the exercises. The researchers were blinded to the recruitment, randomization and assessment procedures which were carried out by an assistant who was blinded to the treatment protocols of the different groups. The research assistant was also credentialed in McKenzie method. The questionnaires used in this study were self- administered.

### Data analysis

Data were analyzed using descriptive of mean and standard deviation; and inferential statistics. One-way ANOVA was used to compare the participants’ general characteristics and pain intensity by treatment groups. Pearson's Product Moment Correlation Analysis was used to test the relationship between HRQoL and intensity of pain. The Kruskal Wallis test was used to compare the treatment outcomes (mean change) on HRQoL across group at week four and eight of the study respectively. Friedman's ANOVA and Wilcoxon signed ranked tests for multiple comparisons were used to compare within group changes in across the three study time points Alpha level was set at p = 0.05. The data analyses were carried out using SPSS 13.0 version software (SPSS Inc., Chicago, Illinois, USA).

## Results

The mean age, height, weight and BMI of all the participants was 51.8 ± 7.35 years, 1.66 ± 0.04m, 76.2±11.2 Kg and 27.2 ± 4.43 kg/m2 respectively. Comparison of the participants’ general characteristics by treatment groups revealed that the participants in the different groups were comparable in their general characteristics (p > 0.05) (Table 1).

**Table 1:** One-way ANOVA comparison of the participants’ general characteristics and pain intensity by treatment groups

	MPG	MPSBEEG	MPDBEEG		
	(n = 25)	(n = 22)	(n = 20)		
**Variable**	x ± SD	x ± SD	x ± SD	F-ratio	p-value
**Age (yr)**	50.6 ± 7.57	51.2 ± 7.50	53.8 ± 6.83	1.106	0.339
**Height (m)**	1.67 ± 0.04	1.66 ± 0.04	1.68 ± 0.04	2.185	0.331
**Weight (Kg)**	76.3 ± 9.95	75.2 ± 13.2	77.2 ± 10.8	0.156	0.856
**BMI (Kg/m2)**	27.5 ± 4.20	27.3 ± 5.25	26.9 ± 3.89	0.093	0.912
**VAS**	6.56 ± 1.83	6.50 ± 1.71	6.60 ± 1.79	0.017	0.983

Alpha level was set at p <0.05

Key: MPG = McKenzie Protocol Group; MPSBEEG = McKenzie Protocol plus Static Back Endurance Exercise Group; MPDBEEG = McKenzie Protocol plus Dynamic Back Endurance Exercise Group; x = Mean; SD = Standard deviation; VAS = Visual Analogue Scale

The mean pain intensity score (VAS) reported by the participants was 6.55 ± 1.75. The relationship between each of the eight domains of HRQoL and intensity of pain (VAS score) is presented in Table 2.

**Table 2:** Relationship between Health-Related Quality of Life and intensity of pain (VAS score) (n = 67)

Domain	r	p-value
**GH**	-0.603	0.001
**PF**	-0.772	0.001
**RP**	-0.759	0.001
**RE**	-0.755	0.001
**SF**	-0.878	0.001
**MH**	-0.86	0.001
**BP**	-0.874	0.001
**EV**	-0.857	0.001

From the result, correlation co-efficient (r) ranged between-0.603 to-0.878 at p = 0.001.

Table 3 shows the comparison of the participants’ baseline measure of HRQoL.

**Table 3:** Kruskal Wallis comparison of the participants’ baseline assessment of HRQoL

	Mean Rank				
Outcome	MPG (n = 25)	MPSBEEG (n = 22)	MPDBEEG (n = 20)	H	p-value
**GH**	33.3	32.9	36.2	0.364	0.839
**PF**	33.0	36.3	32.8	0.442	0.802
**RP**	35.9	32.3	33.0	0.387	0.824
**RE**	34.2	34.7	33.0	0.083	0.96
**SF**	33.4	36.0	32.6	0.351	0.839
**MH**	32.5	35.2	34.5	0.246	0.884
**BP**	36.7	31.6	33.2	0.867	0.648
**EV**	31.7	35.6	35.1	0.597	0.742

The results indicate that the participants in the different treatment groups were comparable in all the domains of HRQoL (p > 0.05). Within-group comparison of HRQoL in MPG, MPSBEEG and MPDBEEG across the 3 time points (weeks 0-4, 4-8 and 0-8) of the study showed that there were significant improvements (p < 0.05) (Table 4). Comparison of treatment outcomes (mean change score (MCS)) at week four and eight of the study are presented in Table 5. There were significant differences in SF-36 scores across the group (p > 0.05) at the end of the 4th and 8th week of the study respectively. The Tukey multiple comparisons post-hoc analysis was used to elucidate where the differences within between groups lie. The result indicated that MPSBEEG and MPDBEEG had significantly higher MCS on all domains of SF-36 compared with MPG at week four and eight respectively (p < 0.05). There was no significant difference between the MPSBEEG and MPDBEEG in the MCS of General Health Perception domain of SF-36 at week four; and on General Health Perception and Physical Functioning Domains of SF-36 at week eight respectively. However, MPDBEE had significantly higher treatment effects on other domains of HRQoL (p = 0.001).

**Table 4:** Friedman's ANOVA and Wilcoxon signed ranked test multiple comparisons of HRQoL among MPG, MPSBEEG and MPDBEEG across the 3 time points of the study

	Mean Rank				
Outcome	Baseline	4th week	8th week	X^2^	p-value
**MPG (n = 25)**					
**GH**	33.3^a^	59.3^b^	69.2^c^	50.00	0.001
**PF**	33.0^a^	57.8^b^	66.8^c^	48.08	0.001
**RP**	35.9^a^	38.5^b^	48.4^c^	45.96	0.001
**RE**	34.2^a^	43.8^b^	52.7^c^	40.735	0.001
**SF**	33.4^a^	64.8^b^	72.6^c^	48.91	0.001
**MH**	32.5^a^	56.4^b^	63.1^b^	47.446	0.001
**BP**	36.7^a^	54.4^b^	66.2^c^	52.108	0.001
**EV**	31.7^a^	57.7^b^	64.8^c^	47.265	0.001
**MPSBEEG (n = 22)**					
**GH**	32.9^a^	64.4^b^	72.5^c^	42.34	0.001
**PF**	36.3^a^	65.0^b^	74.0^c^	44.5	0.001
**RP**	32.3^a^	48.4^b^	59.2^c^	41.4	0.001
**RE**	34.7^a^	49.6^b^	57.3^c^	39.321	0.001
**SF**	36.0^a^	72.3^b^	81.2^c^	43.517	0.001
**MH**	35.2^a^	59.9^b^	68.1^c^	42.091	0.001
**BP**	31.6^a^	61.3^b^	70.2^c^	44.12	0.001
**EV**	35.6^a^	60.7^b^	71.2^c^	38.273	0.001
**MPDBEEG (n = 20)**					
**GH**	36.2^a^	67.6^b^	77.4^c^	38.4	0.001
**PF**	32.8^a^	72.8^b^	80.4^c^	40.22	0.001
**RP**	33.0^a^	55.2^b^	65.0^c^	44.11	0.001
**RE**	33.0^a^	53.4^b^	62.1^c^	42.34	0.001
**SF**	32.6^a^	72.7^b^	81.6^c^	39.519	0.001
**MH**	34.5^a^	67.5^b^	76.4^c^	43.124	0.001
**BP**	33.2^a^	65.0^b^	71.2^c^	42.645	0.001
**EV**	35.1^a^	66.2^b^	78.4^c^	45.6	0.001

**Table 5:** Kruskal Wallis comparison of the participants’ treatment outcomes (mean change) at week four of the study

	Mean rank				
	MPG (n = 25)	MPSBEEG (n = 22)	MPDBEEG (n = 20)	H	p-value
**Week 4**					
**GH**	19.0^a^	41.7^b^	44.3^b^	24.06	0.001
**PF**	18.1 ^a^	37.1^b^	50.5^c^	31.887	0.001
**RP**	14.8^a^	40.1^b^	51.3^c^	42.277	0.001
**RE**	22.5^a^	36.7^b^	45.4 ^c^	16.702	0.001
**SF**	22.4^a^	39.5^b^	42.4 ^c^	14.397	0.001
**MH**	21.7^a^	30.5^b^	53.3 ^c^	30.639	0.001
**BP**	18.9^a^	38.4^b^	48.1 ^c^	26.813	0.001
**EV**	23.4 ^a^	36.0^b^	45.1^b^	14.193	0.001
**Week 8**					
**GH**	22.1^a^	43.2^b^	44.1^b^	27.01	0.001
**PF**	19.2^a^	40.1^b^	52.3^b^	33.122	0.001
**RP**	16.4 ^a^	41.2^b^	51.7^c^	46.108	0.001
**RE**	23.7^a^	38.2^b^	46.3^c^	22.112	0.001
**SF**	24.2^a^	40.4^b^	44.0^c^	16.014	0.001
**MH**	23.4^a^	36.2^b^	53.1 ^c^	36.114	0.001
**BP**	21.3^a^	40.3^b^	49.2 ^c^	28.612	0.001
**EV**	24.7 ^a^	38.2^b^	47.0^b^	15.018	0.001

## Discussion

This study evaluated the relationship between HRQoL and pain intensity, and the influence of static and dynamic back extensors’ endurance exercises on HRQoL in Nigerian patients with LMLBP treated with the MP. The mean age of the patients in this study was 51.8 ± 7.35 years. This age falls within the age bracket during which LBP is reported to be a more common problem [55]. From the result of this study, no significant difference in physical characteristics and pain intensity was found in the different treatment groups at baseline. Baseline characteristics are believed to be predictors of response to treatment in clinical trials for LBP [56]. Comparability in baseline measure in clinical trials is reported to reduce the chances of co-founders other than the intervention in predicting outcomes. Therefore, it is implied that the results obtained at different point in the course of this study could have been largely due to the effects of the various treatment regimens.

This study investigated the relationship between HRQoL and the intensity of pain. From the result, significant moderate to high inverse relationships were found between pain intensity and the different domains of HRQoL. General health perception showed the least correlation (r = -0.603; p = 0.001) while social functioning had the highest correlation with pain intensity (r = -0.878; p = 0.001). It is inferred from the study's result that HRQoL of patients with long-term LBP decreases with severity of pain. Previous studies have reported an association between LBP and psychosocial factors [26, 57]. Specifically, significant inverse correlation has been reported between severity of pain and quality of life in patients with chronic LBP [57–59]. Pain is believed to have a profound effect on HRQoL [59] and the degree, to which the patients believe that they are disabled by it, is a powerful factor in the extent of their quality of life impairments [60]. Therefore, quality of life is an indicator of the level of endurance of people to pain [61].

Within-group comparison of each of MP, MP plus Static Back Endurance Exercise (MPSBEE) and MP plus Dynamic Back Endurance Exercise (MPDBEE) across the 3 time-points (weeks 0-4, 4-8 and 0-8) of the study revealed that each treatment regimen led to significant improvement in HRQoL. Patients in this study displayed baseline values of the SF-36 comparable to those described in other studies on chronic LBP [62]. The baseline values of all domains of the SF-36 observed in this study were lower than those of adult normative data reported by Jenkinson et al [63] leaving room for any improvement accruable to treatment regimens to be assessed. From this study, all the eight domains of the SF-36 significantly improved at the 4th and 8th week assessment. However, on the final assessment, social functioning, general health perception and bodily pain improved more than the other domains of SF-36 in the MPG. General health perception, physical functioning, social functioning, bodily pain and energy vitality improved more than the other domains of SF-36 in the MPSBEEG while general health perception, physical functioning, social functioning, bodily pain and energy vitality improved more than the other domains of SF-36 in the MPDBEEG. Role physical, role emotional and mental health were the least improved domains of the SF-36 among the treatment groups. Though significant improvements were observed in the different domains by treatment groups on final assessment, the values were still lower than the adult normative data for general health status assessed using the SF-36 questionnaire [63]. A previous study by Smeets and colleagues [64] found that active physical therapy regimen primarily designed to improve physiological aspects of LBP such as aerobic fitness level, low back muscle strength and endurance can also reduce the impact of psychosocial factors that it did not deliberately target. In view of current evidence, Hill and Fritz [57] suggest that it may not necessarily follow that a psychologist is better placed to improve treatment outcomes than a physical therapist, even when a goal of treatment is the mediation of a psychosocial factor. Hill and Fritz [57] also argue that psychosocial factors including fear of movement, anxiety, a faulty coping strategy and quality of life have a strong influence on the success of treatment for patients with back pain at a group level. Literature suggests that exercise generally has a potential benefit on psychosocial aspect of patient with long-term LBP. Long-term LBP leads to deconditioning [65] and many problems associated with deconditioning are believed to be reversible through general and specific exercise regimens [66]. Harding and Watson [66] note that improvement in overall physical function is linked with improvement in psychosocial function. Unfortunately, there is a dearth of studies on the effect of the MP and back extensors endurance exercises on HRQoL in patients with long-term mechanical LBP.

From the result of this study, comparison of the different treatment regimens indicate that MPSBEE and MPDBEE had significantly higher treatment effect on all domains of HRQoL compared with MP at week four and eight respectively. MPSBEE and MPDBEE were comparable in their effect on general health perception domain at week four; and on health perception and physical functioning domains of the HRQoL at week eight. However, MPDBEE had significantly higher treatment effects on other domains of HRQoL. Generally, exercise seems to leads to improved wellness and quality of life. Still, there does not appear to be a consensus of opinion on the most effective programme designed to maintain exercise benefits. The McKenzie method is a popular and promising classification-based treatment for LBP among physical therapists [3] in addition to delivering theoretical information in order to educate patients about their condition, so that patients are better able to understand their condition and how to change their behaviour towards an episode of LBP [67]. However, few studies have investigated the effect of the MP on HRQoL in patients with LMLBP. Udermann et al [68] found significant improvements in HRQoL measures in chronic LBP patients treated with MP but reported that the addition of resistance training for the lumbar extensors provided no additional benefit. In recent times, endurance training of the low-back extensors aimed at improving physical performance and psychosocial health in patients with LBP has increased in popularity [69, 48, 52, 70], yet their effectiveness in enhancing quality of life remains unclear [71].

The observed efficacy of the MP, MPSBEE and MPDBEE in this study could be as a result of the fact that each of the regimen contained active exercise carried out in extension positions. Active exercise can be described as functional exercise performed by the patient or client. Previous studies have shown that active exercise, irrespective of the type is more effective in the management of patients with long-term LBP than passive therapy [72, 73]. The MP utilizes a system of patient self generated force to mobilize or manipulate the spine through a series of active repeated movements or static positioning and it is based on the patient's pain response to certain movements and postures during assessment [3]. Similarly, endurance exercises are active exercises that require static posturing or repeated movements in order to initiate overload stimuli on the musculature. The different treatment regimen in this study had movement components, either from the MP which is the baseline treatment for all the groups or from the back extensors endurance exercise protocols. It is postulated from the results of this study that the significant higher treatment outcome of MPDBEE might be due to the combined effects of movements and overload stimulus on the back extensor muscles. MPDBEE seems to contain movement ingredients, firstly, from the MP which is the baseline treatment for this group and it involved a series of active repeated movements. Secondly, the dynamic back extensors endurance exercise also involved repeated movements of the trunk and limbs in the sagittal plane. It seems that extension exercise with movement elements carried out in patterns similar to the daily tasks motions might help to improve psychosocial aspects of long-term LBP as observed in this study.

### Limitations of the study

The generalizability of the findings of this study is limited by the fact that a generic quality of life tool was employed because of the scarcity of standard HRQoL tools with documented psychometric properties specific for patients with LBP. Theoretically, specific HRQoL measures are opined to be more responsive than generic HRQL measures [74]. Like all other self-reported assessment, it is possible that the patients in this study might have given exaggerated responses or overestimated the effect of exercise on their HRQoL. Furthermore, individuals’ perception of psychosocial construct such as HRQoL is believed to be influenced by subjective interpretation and cultural bias [75, 76]. The high drop-out rate observed in this study is also a potential limitation and source of bias which may limit the interpretation and generalizability of study results. Finally, the treatment outcomes of the different regimens were only measured over such a short period of time of eight weeks.

## Conclusion

Health-related quality of life of patients with long-term LBP decreases with severity of pain. The McKenzie Protocol, static and dynamic back extensors endurance exercises had significant therapeutic effect on HRQoL in patients with LMLBP. However, the addition of dynamic back extensors endurance exercise to MP led to higher improvement on HRQoL. It is recommended that static or dynamic endurance exercise be combined with MP in patients with LMLBP to derive maximum improvement in general health status.

## References

[1] Waddell G (1998). The back pain revolution.

[2] Burton AK, Balague F, Cardon G, Eriksen HR, Henrotin Y, Lahad A (2006). On behalf of the COST B13 Working Group on Guidelines for Prevention in Low Back Pain. European guidelines for prevention in low back pain - November 2004. Eur Spine J..

[3] Mckenzie RA (1990). Treat Your Own Back. Spinal Publication.

[4] Sikorski JM, Stampfer HG, Cole RM, Wheatley AE (1996). Psychological aspects of chronic low back pain. Aust N Zeal J Surg..

[5] Filho IT, Simmonds MJ, Protas EJ, Jones S (2002). Back pain, physical function, and estimates of aerobic capacity: what are the relationships among methods and measures?. Am J Phys Med Rehabil..

[6] Anderson GBJ (1999). Epidemiologic features of chronic low-back pain. Lancet..

[7] World Health Organization (WHO) Scientific Group on the Burden of Musculoskeletal Conditions of the Start of the New Millennium (2003). The burden of musculoskeletal conditions at the start of the new millennium.

[8] Louw QA, Morris LD, Grimmer-Somers K (2007). The prevalence of low back pain in Africa: a systematic review. BMC Musculoskelet Disord..

[9] van Tulder MW, Koes BW, Bouter LM (1997). Conservative treatment of acute and chronic nonspecific low back pain. A systematic review of randomized controlled trials of the most common interventions. Spine..

[10] Quittan M (2002). Management of Back Pain. Disabil Rehabil..

[11] Bigos SJ, McKee J, Holland JP, Holland CL, Hildebrandt J (2001). Back pain; the uncomfortable truth-assurance and activity paradigm. Der Schmertz..

[12] Deyo RA, Tsui-Wu YJ (1987). Functional disability due to low-back pain: a population-based study indicating the importance of socioeconomic factors. Arthritis Rheum..

[13] Coste J, Delecoeuillerie G, Cohen de Lara A, Le Parc JM, Paolaggi JB (1994). Clinical course and prognostic factors of acute low-back pain: an inception cohort study in primary care practice. BMJ.

[14] Picavet HS, Schouten JS (2003). Musculoskeletal pain in the Netherlands: prevalences; consequences and risk groups; the DMC 3-study. Pain..

[15] Tuzun EH (2007). Quality of life in chronic musculoskeletal pain. Best Pract Res Clin Rheumatol..

[16] Last AR, Hulbert K (2009). Chronic Low Back Pain: Evaluation and Management. Am Fam Physician. http://www.vertebrologi.ru/biblio/chronic_back.pdf.

[17] Linton SJ (2000). A review of psychological risk factors in back and neck pain. Spine..

[18] Scholich SL, Hallner D, Wittenberg RH, Hasenbring MI, Rusu AC (2012). The relationship between pain, disability, quality of life and cognitive-behavioural factors in chronic back pain. Disabil Rehabil..

[19] Geisser ME, Robinson ME, Miller QL, Bade SM (2003). Psychosocial factors and functional capacity evaluation among persons with chronic pain. J Occup Rehabil..

[20] Lamé IE, Peters ML, Vlaeyen JW, Kleef M, Patijn J (2005). Quality of life in chronic pain is more associated with beliefs about pain, than with pain intensity. Eur J Pain..

[21] Deyo RA, Andersson G, Bombardier C, Cherkin DC, Keller RB, Lee CK (1994). Outcome measures for studying patients with low back pain. Spine..

[22] Bombardier C (2000). Outcome assessments in the evaluation of treatment of spinal disorders. Spine..

[23] Ware JE, Snow KK, Kosinski M, Gandek B (1993). SF-36 Health Survey - Manual and Interpretation Guide. Boston: The Health Institute; New England Medical Center..

[24] Ware JE, Sherbourne CD (1992). The MOS 36-item shortform health survey (SF-36) I. Conceptual framework and item selection. Med Care..

[25] Main CJ, George SZ (2011). Psychosocial Influences on Low Back Pain: Why Should You Care?. Phys Ther..

[26] Vlaeyenm JWS, Kole-Snijders AM, Boeren RG, van Eek H (1995). Fear of movement/(re)injury in chronic low back pain and its relation to behavioral performance. Pain..

[27] Gatchel RJ, Polatin PB, Mayer TG (1995). The dominant role of psychosocial risk factors in the development of chronic low back pain disability. Spine..

[28] George SZ, Joel E Bialosky, Julie M Fritz (2004). Beliefs Acute Low Back Pain and Elevated Fear-Avoidance Physical Therapist Management of a Patient With. Phys Ther..

[29] Hägg O, Burckhardt C, Fritzell C, Nordwall A (2003). Quality of Life in Chronic Low Back Pain: A Comparison with Fibromyalgia and the General Population. J Muscoskel Pain..

[30] Woby SR, Watson PJ, Roach NK, Urmston M (2004). Are changes in fear-avoidance beliefs, catastrophizing, and appraisals of control, predictive of changes in chronic low back pain and disability?. Eur J Pain..

[31] Weiner BK (2008). Spine Update - The Biopsychosocial Model and Spine Care. Spine..

[32] Lopez A, Mathers C, Ezzati M, Jamison D, Murray J (2006). Global and regional burden of disease and risk factors, : Systematic analysis of population health data 2001. Lancet..

[33] Australian Bureau of Statistics (ABS) (2006). Physical activity in Australia: a snapshot, 2004-05. ABS cat. no. 4835.0.55.001.

[34] Cavill N, Kahlmeier S, Racioppi F Physical activity and health in Europe: evidence for action. http://www.euro.who.int/en/publications/abstracts/physical-activity-and-health-in-europe-evidence-for-action.

[35] Centers for Disease Control and Prevention (CDCP) (2013). Exercise or Physical Activity. http://www.cdc.gov/nchs/fastats/exercise.htm.

[36] Hayden JA, van Tulder MW, Tomlinson G (2005). Systematic Review: Strategies for using exercise therapy to improve outcomes in chronic low-back pain. Ann Int Med..

[37] Brukner P, Khan K (1993). Clinical Sports Medicine.

[38] Cherkin DC, Deyo RA, Battla MC, Street JH, Hund M, Barlow W (1998). A comparison of Physical therapy chiropractice manipulation or an educational booklet for the treatment of low back pain. New Eng J Med..

[39] McKenzie R, May S (2003). The lumbar spine. Mechanical diagnosis & therapy.

[40] Machado LA, de Souza MS, Ferreira PH, Ferreira ML (2006). The McKenzie method for low back pain: a systematic review of the literature with a meta-analysis approach. Spine..

[41] Ayanniyi O, Lasisi OT, Adegoke BOA, Oni-Orisan MO (2007). Management of low back pain: Attitudes and treatment preferences of physiotherapists in Nigeria. Afr J Biomed Res..

[42] Mbada CE, Ayanniyi O, Ogunlade SO (2011). Effect of static and dynamic back extensor muscles endurance exercise on pain intensity, activity limitation and participation restriction in patients with long-term mechanical low-back pain. Med Rehabil..

[43] Cohen J (1988). The analysis of variance and covariance: Sample size tables. In Statistical Power Analyses for Behavioural Sceinces 2nd Ed Chapter 8..

[44] Bronfort G, Bouter LM (1999). Responsiveness of general health status in chronic low back pain: a comparison of the COOP charts and the SF-36. Pain..

[45] Taylor SJ, Taylor AE, Foy MA, Fogg AJB (2001). Responsiveness of common outcome measures for patients with low back pain. Spine..

[46] Jensen MP, McFarland CA (1993). Increasing the reliability and validity of pain intensity measurement in chronic pain patients. Pain..

[47] Von Korff M, Deyo RA, Cherkin D, Barlow SF (1993). Back pain in primary care: Outcomes at 1 year. Spine.

[48] Moffroid MT, Haugh LD, Haig AJ, Henry SM, Pope MH (1993). Endurance training of trunk extensor muscles. Phys Ther..

[49] Adegoke BOA, Babatunde FO (2007). Effect of an exercise protocol on the endurance of trunk extensor muscles: a RCT. Hong Kong Physiother J..

[50] Petrofsky JS, Lind AR (1975). Aging, isometric strength and endurance; and cardiovascular responses to static effort. J Appl Physiol..

[51] Bonde-Petersen F, Mork AL, Nielsen E (1975). Local muscle blood flow and sustained contractions of human arm and back muscles. Eur J Appl Physiol Occup Physiol..

[52] Chok B, Lee R, Latimer J, Beng Tan S (1999). Endurance training of the trunk extensor muscles in people with sub acute low back pain. Phys Ther..

[53] Fox EL, Bowers RW, Foss ML (1988). The physiological basis of physical education and athletics.

[54] Liddle SD, Baxter GD, Gracey JH (2004). Exercise and chronic low back pain - what works?. Pain..

[55] Leboeuf-Yde C, Kyvik KO (1998). At what age does low back pain become a common problem? A study of 29;4 24 individuals aged 12-41 years. Spine..

[56] Underwood MR, Morton V, Farrin A, UK BEAM trial team (2007). Do baseline characteristics predict response to treatment for low back pain? Secondary analysis of the UK BEAM dataset. Rheumatology..

[57] Hill JC, Fritz JM (2011). Psychosocial influences on low back pain; disability; and response to treatment. Phys Ther..

[58] Sengul Y, Kara B, Arda MN (2010). The relationship between health locus of control and quality of life in patients with chronic low back pain. Turk Neurosurg..

[59] Tavafian SS, Eftekhar H, Mohammad K, Jamshidi AR, Montazeri A, Shojaeezadeh D, Ghofranipour F (2005). Quality of Life in Women with Different Intensity of Low Back Pain. Iran J Public Health..

[60] Turner JA, Jensen MP, Romano JM (2000). Do beliefs, coping, and catastrophizing independently predict functioning in patients with chronic pain. Pain..

[61] Lyons RA, Lo SV, Littlepage BNC (1994). Comparative health status of patients with 11 common illnesses in Wales. J Epidemiol Community Health..

[62] Lurie J (2000). A review of generic health status measures in patients with low back pain. Spine.

[63] Jenkinson C, Coulter A, Wright L (1993). Short form 36 (SF 36) health survey questionnaire: normative data for adults ofworking age. BMJ..

[64] Smeets RJ, Vlaeyen JW, Kester AD, Knottnerus JA (2006). Reduction of pain catastrophizing mediates the outcome of both physical and cognitive-behavioral treatment in chronic low back pain. J Pain..

[65] Verbunt JA, Seelen HA, Vlaeyen JW, van de Heijden GJ, Heuts PH, Pons K, Knottnerus JA (2003). Disuse and deconditioning in chronic low back pain: concepts and hypotheses on contributing mechanisms. Eur J Pain..

[66] Harding VR, Watson PJ (2000). Increasing Activity & Improving Function In Chronic Pain Management. Physiotherapy..

[67] Garcia AN, Gondo FLB, Costa RA, Cyrillo FN, Silva TM, Costa LCM, Costa LOP (2011). Effectiveness of the back school and McKenzie techniques in patients with chronic non-specific low back pain: a protocol of a randomised controlled trial. BMC Musculoskelet Disord..

[68] Udermann BE, Mayer JM, Donelson RG, Graves JE, Murray SR (2004). Combining lumbar extension training with McKenzie therapy: Effects on pain; disability; and psychosocial functioning in chronic low back pain patients. GLMJ..

[69] Kovascs FM, Abraira V, Zamora J, Fernandez C (2005). The transition from acute to subacute and chronic low back pain: A study based on determinants of quality of life and prediction of chronic disability. Spine..

[70] Johnson OE, Adegoke BOA, Ogunlade SO (2010). Comparison of four physiotherapy regimens in the treatment of long-term mechanical low back pain. JJPTA..

[71] Shaughnessy M, Caulfield B (2004). A pilot study to investigate the effect of lumbar stabilisation exercise training on functional ability and quality of life in patients with chronic low back pain. Int J Rehabil Res..

[72] Kankäänpää M, Taimela S, Airaksien OJ, Hannnien O (1999). The efficacy of active rehabilitation in chronic low back pain. Effect on pain intensity; self-experienced disability and lumbar fatigability. Spine..

[73] Rainville J, Hartigan C, Martinez E, Limke J, Jouve C, Finno M (2004). Exercise as a treatment for chronic low back pain. Spine J..

[74] Guyatt Gordon (1997). Insights and Limitations from Health-Related Quality-of-Life Research. Gen Intern Med..

[75] Kleinman A, Eisenberg L, Good B (1978). Culture, illness and care: clinical lessons from anthropologic and cross-cultural research. Ann Intern Med..

[76] Carr AJ, Higginson IJ (2001). Are quality of life measures patient centred?. BMJ..

